# Capture and commercialization of blue land crabs ("guaiamum") *Cardisoma guanhumi *(Lattreille, 1825) along the coast of Bahia State, Brazil: an ethnoecological approach

**DOI:** 10.1186/1746-4269-8-12

**Published:** 2012-03-19

**Authors:** Angélica MS Firmo, Mônica MP Tognella, Saulo R Silva, Raynner RRD Barboza, Rômulo RN Alves

**Affiliations:** 1 Departamento de Ciências Agrárias e Biológicas, Programa de Pós Graduação em Biodiversidade Tropical (Ecologia), Universidade Federal do Espírito Santo/Centro Universitário Norte do Espírito Santo - UFES/CEUNES, São Mateus, Brazil; 2 Departamento de Biologia, Programa de Pós-Graduação em Ciências Biológicas (Zoologia) Universidade Estadual da Paraíba - UEPB, Paraíba, Brazil

**Keywords:** Crab harvesters, Environmental perception, Mangrove, Conservation

## Abstract

**Background:**

Blue Land Crab (*Cardisoma guanhumi*) is one of the most important crustacean species captured and commercialized in Brazil. Although this species is not considered to be threatened with extinction, populations of *C. guanhumi *are known to be rapidly diminishing due to heavy harvesting pressures and degradation of their natural habitats, highlighting the necessity of developing and implanting management and protection strategies for their populations. There have been no ethnozoological publications that have focused specifically on *C. guanhumi*, in spite of importance of this type of information for developing efficient management plans of resource utilization. So, the present work describes the ethnoecological aspects of the capture and commercialization of *C. guanhumi *by a fishing community in northeastern Brazil.

**Methods:**

Field work was carried out in the municipality of Mucuri, Bahia in Brazil, between the months of January and March/2011 through the use of open semi-structured interviews with all of the crustacean harvesters in city who acknowledged their work in capturing this species, totaling 12 interviewees. The informants were identified through the use of the "snowball" sampling technique. In addition to the interviews themselves, the "guided tour" technique and direct observations was employed.

**Results:**

According all the interviewees, the *C. guanhumi *is popularly called "guaiamum" and is collected in "apicum" zones. They recognize sexual dimorphism in the species based on three morphological characteristics and the harvesters also pointed two stages in the reproductive cycle during the year and another phase mentioned by the interviewees was ecdysis. All of the interviewed affirmed that the size and the quantities *C. guanhumi *stocks in Mucuri have been diminishing. All of the interviewees agreed that the species and other mangrove resources constituted their principal source of income. The harvesters dedicated three to five days a week to collect Blue Land Crabs and the principal technique utilized for capturing is a trap called a "ratoeira" (rat-trap).

**Conclusions:**

The results of the present work demonstrated that the community retains a vast and important volume of knowledge about *C. guanhumi *that could subsidize both scientific studies and the elaboration of viable management and conservation strategies for this species.

## Background

Mangrove ecosystems are restricted to intertidal coastal zones in tropical and subtropical regions. These transitional environments are characterized by high levels of primary productivity and high capacities for transforming nutrients into organic material [1]. Mangrove swamps are encountered along almost the entire Brazilian coast from the Oiapoque River in the state of Amapá in the north to Laguna, in the state of Santa Catarina [2] and they occupy a total area of approximately 25,000 Km^2 ^[3]. The ecological services furnished by mangrove swamps are quite numerous and include protecting against coastal erosion, preventing inland flooding, and maintaining coastal biodiversity [4].

In addition to their ecological importance, mangrove swamps furnish a wide spectrum of natural products to human populations, such as: wood, fish [5], crustaceans, mollusks, dyes [6], charcoal, tannins, and plant medicines [7], and coastal communities throughout the world depend on these areas for their subsistence [3,8,9]. Brachyura crustaceans are one of the most economically important resources of estuarine communities in Brazil [1,10,11].

Among the most important crustacean species captured and commercialized in Brazil are: the Blue Land Crab ("goiamum") (*Cardisoma guanhumi*), the Mangrove Root Crab ("aratu") (*Goniopsis cruentata*), the Swimming Crab group ("siris") (*Callinectes *spp.), and the Mangrove Crab ("caranguejo-uçá") (*Ucides cordatus*) [12,13]. The Blue Land Crab is a semi-terrestrial species that lives in mangroves above the high tide line [14,15] in Brazil between the states of Ceará and Santa Catarina [14,16] and is of significant economic importance in the northeastern region of that country. Although this species is not considered to be threatened with extinction, populations of *C. guanhumi *are known to be rapidly diminishing due to heavy harvesting pressures and degradation of their natural habitats [17,18] - which has motivated their inclusion on the National List of invertebrate aquatic and fish species that are threatened, over-exploited, or threatened with over-exploitation (Normative Instruction n° 5, of 21 of May of 2004) [19].

The harvesting pressure felt by this crustacean as well as the ongoing degradation of their habitat both highlight the necessity of developing and implanting management and protection strategies for their populations. These efforts will in turn require additional research on the biological aspects of this species as well as ethnoecological investigations directed towards the socioeconomic aspects of harvesting activities. The few published works available about this species deal mostly with its occurrence and distribution [16,20-23]. A recent ethnozoological review by Alves and Souto [24] listed a total of 23 publications focusing on ethnocarcinology in Brazil, most of them dealing with the species *Ucides cordatus *(Swamp Ghost Crab) [6,25-31]. There have been no ethnozoological publications that have focused specifically on *Cardisoma guanhumi*, in spite of importance of this type of information for developing efficient management plans of resource utilization [32-36]. Information about the biology of this species associated with the accumulated empirical knowledge of the human populations that use these resources can furnish important subsidies to improve regulations designed to rationalize the harvesting and management of natural stocks of this species.

The present work describes the ethnoecological aspects of the capture and commercialization of *C. guanhumi *by a fishing community in northeastern Brazil. It is designed to provide a better understanding of the environmental perception of the fishermen there within their own socioeconomic context and to provide subsidies for the establishment of social programs that can better the lives of these workers and simultaneously aid in conserving this valuable natural resource.

## Methods

### Study area

**Figure 1:**
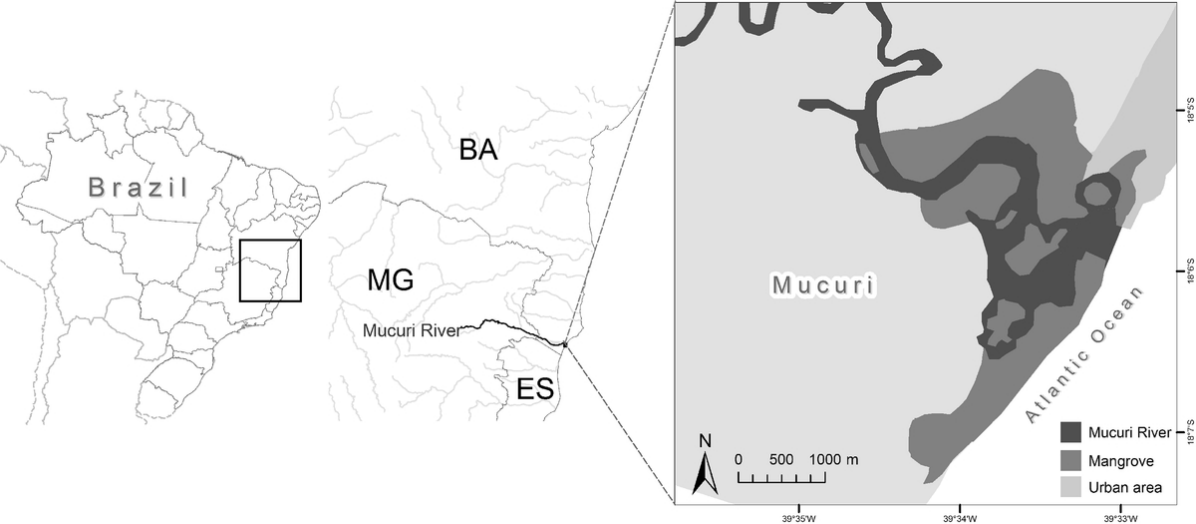
**Map of the Mucuri River estuary, Bahia state, Brazil**.

Field work was carried out in the municipality of Mucuri, in the extreme southern part of the state of Bahia (18° 05'46" S, 39° 33'13 "W) (Figure 1). The region is composed of coastal plains covered by generally low vegetation, with dense "restingas" (sandy coastal areas) and small remnant patches of Atlantic Forest, as well as an exuberant mangrove swamp [37]. Many of the residents of Mucuri make their living from fishing and harvesting crustaceans in this mangrove area [38].

### Data collection and analyses

Information related to ethno-carcinological knowledge about *C. guanhumi *was obtained through the use of open semi-structured interviews with all of the crustacean harvesters in Mucuri who acknowledged their work in capturing this species, totaling 12 interviewees. We elaborated 29 questions dealing with their ecological and biological knowledge of these animals as well as the techniques used in harvesting, storing, and commercializing the crabs. The informants were identified through the use of the "snowball" sampling technique, in which interviewees are chosen based on the recommendations of other informants [39].

Of the twelve crab-harvesters interviewed, all were men between 18 and 51 years old (average 30). Their work experience as harvesters varied from 5 to 35 years (average 22 years).

In addition to the interviews themselves, the "guided tour" technique [40] was employed in excursions into the interior of the Mucuri mangrove accompanied by fishermen to make first hand observations of the activities involved in capturing the crabs. As many questions could not be answered solely through the use of questionnaires, this information was complemented through direct observations (equivalent to the non-member participant observer technique) [41] that allowed close contact between the researcher and the group being studied and facilitated a better comprehension of the information presented [42].

The data was analyzed qualitatively, considering all of the information presented by the interviewees [43]. Two control techniques were adopted to confirm the validity of the information: synchronic situations, in which the same question was presented to different people at approximately the same time; and diachronic situations, in which the same question was submitted to the same individual at distinct time intervals [44].

The present study was approved by the ethics and research committees of the Federal University of Espírito Santo/University Center North of Espírito Santo (UFES/CEUNES). All of the interviews were undertaken between the months of January and March/2011 during previously arranged visits to the residences of the fishermen, or in previously determined alternative localities. Photographic records were made whenever possible, and field data was recorded digitally and subsequently transcribed in the form of texts or tables. The interviews were always preceded by the interviewer's identification with a brief explanation about the purpose of work and a written consent and permission for publication of the images were given by those interviewed.

## Results and discussion

### Ethno-carcinological knowledge

The Blue Land Crab, *Cardisoma guanhumi*, is popularly called "guaiamum" by fishermen and residents of Mucuri, although other regional variations of the term are used in other coastal localities, such as "guaiamu" or "goiamum" [36].

**Figure 2:**
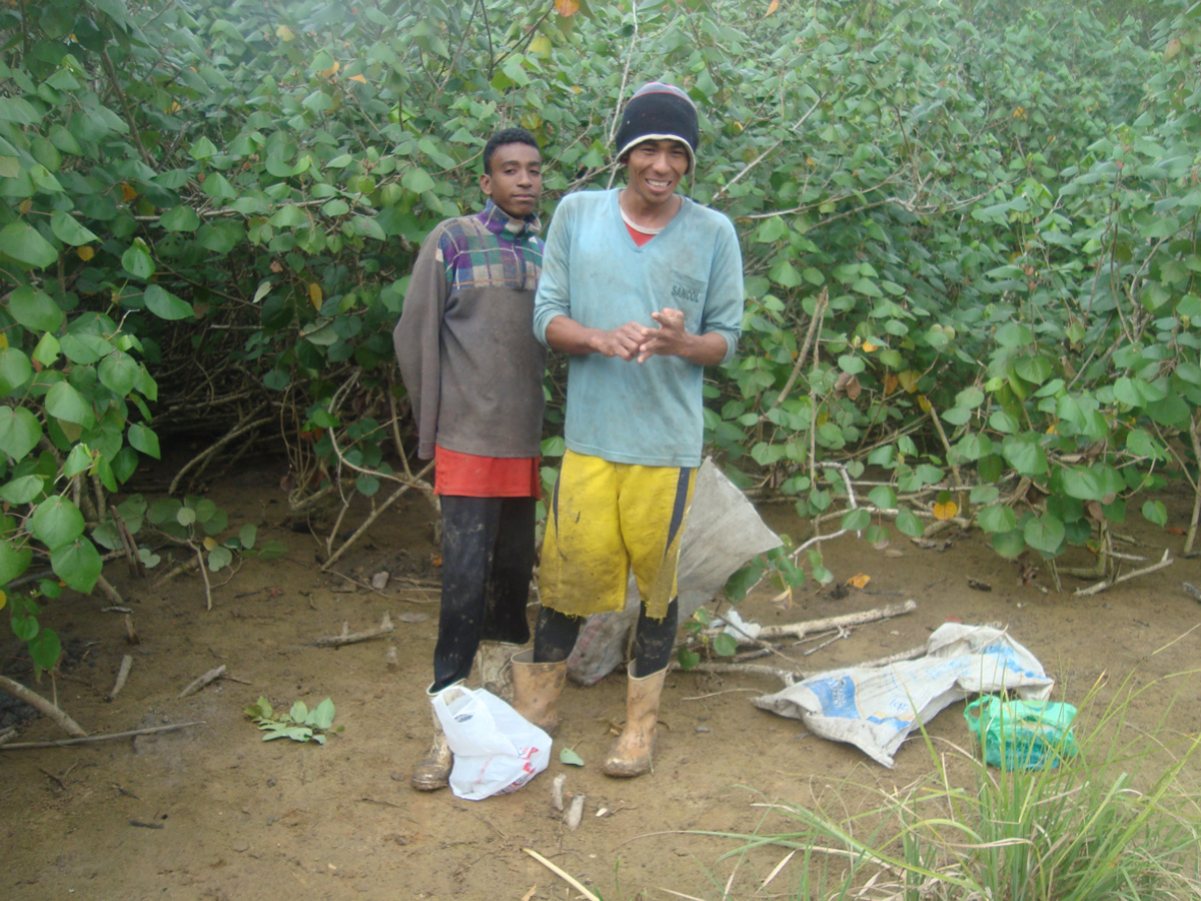
**Crab Collectors in an "apicum" zone of the Mucuri mangrove**.

According all the interviewees in the research area, this crustacean is collected in "apicum" zones (Figure 2), which Soares [45] described as corresponding to generally flat areas with high salinity and acidic levels located above the high-tide line and lacking vegetation (or with only sparse, low growth). The information provided by the local fishermen agreed with published accounts that indicate that Blue Land Crabs normally occupy higher regions of the mangrove swamp above the high-tide line, where the terrain is usually more sandy [46].

The interviewees indicated the existence of two basic types of mangrove areas: "enxuto" (dry) sites where Blue Land Crabs are found, and the "mole" (soft) sites that are preferred by the Mangrove Crab, *Ucides cordatus*. The latter species is known to inhabit galleries carved into soft substrates in the intermediate zone between the high and low tides [30], while the Blue Land Crabs carve their tunnels into higher, sandy ground, while maintaining a small amount of water in bottom of its den. According to the interviewees, Blue Land Crabs normally construct their galleries in the "apicum" zone where the vegetation is distinct from that encountered in the mangrove swamp itself [47].

**Figure 3:**
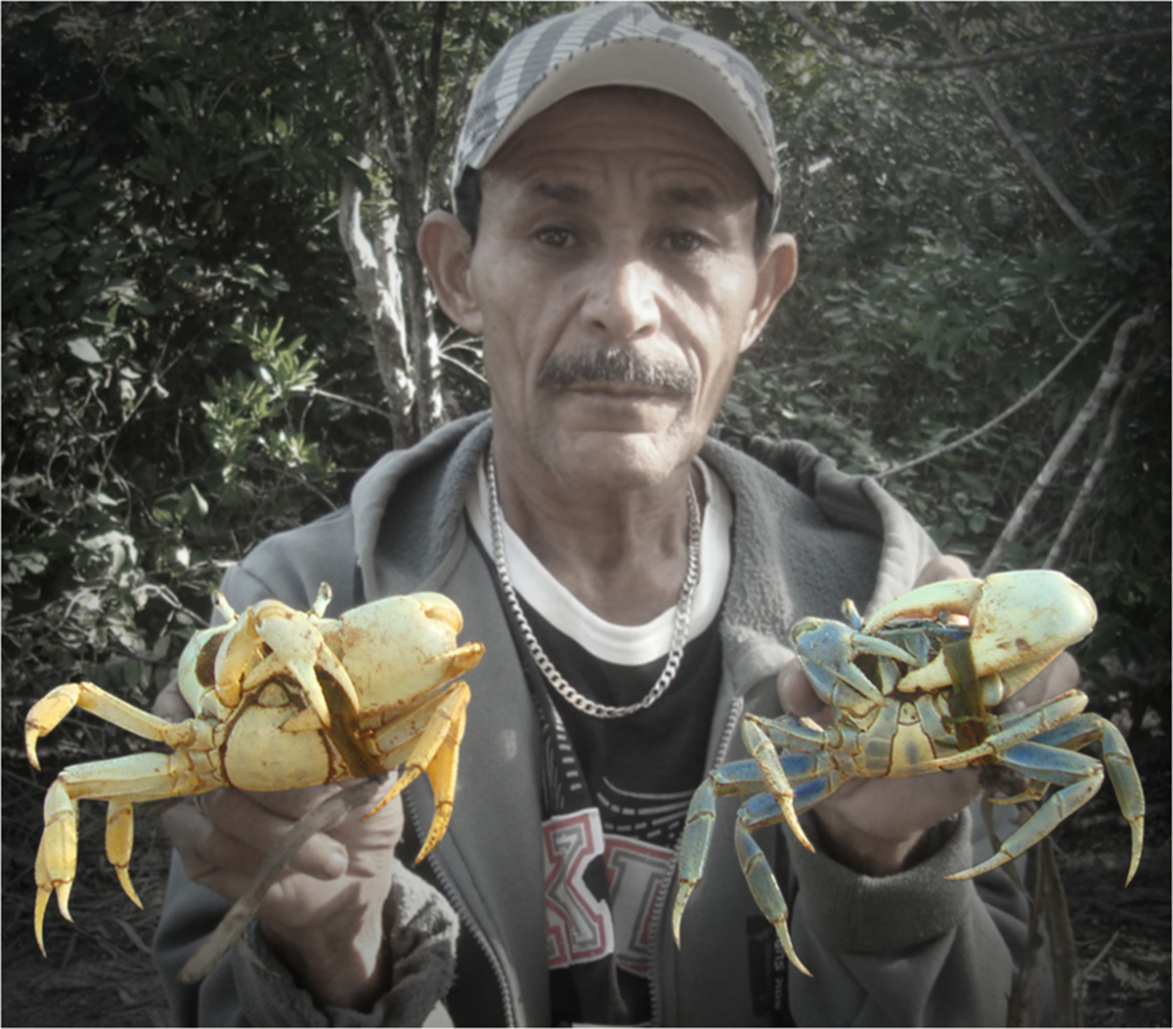
**Collector showing sexual dimorphism of the species *C. guanhumi***.

The harvesters recognized sexual dimorphism in the species based on three morphological characteristics. The first is related to differences in the shapes and sizes of the abdomen (which they call the "imbigo"). The males have thinner and smaller abdomens, while those of the females are larger and wider (and are designated "Apupê") (Figure 3) and are used to carry their egg-masses [48,49]. The same sexual differentiation based on abdomen size was described in studies undertaken in other northeastern mangrove swamps involving the species *C. guanhumi *and *U. cordatus *[50-52]. This type of sexual differentiation was also observed among Swimming Crabs (*Callinectes *spp.) [53], Mangrove Root Crabs (*Goniopsis cruentata*) [54], and Mangrove Crabs [12,38,55,56]. The second morphological difference between the sexes cited by the interviewees was related to their coloration, which could be bluish or purple in males and white in females. According to Gifford [57], young individuals of *C. guanhumi *are purple; the males become bluish or grey-blue when older, while the females become yellow-orange or whitish. These observations were corroborated by the crustacean harvesters. It must be noted, however, that both sexes can become notably bluish (or exhibit brownish or whitish variations) during ecdysis (molting) [55]. Size was the third factor of sexual differentiation pointed out by the fishermen, with the males being generally larger than the females. This difference, in addition to being common among crustaceans, can also occur in response to factors such as reproductive migration, behavioral variations, and differences in life phases or mortality rates [58,59].

**Figure 4:**
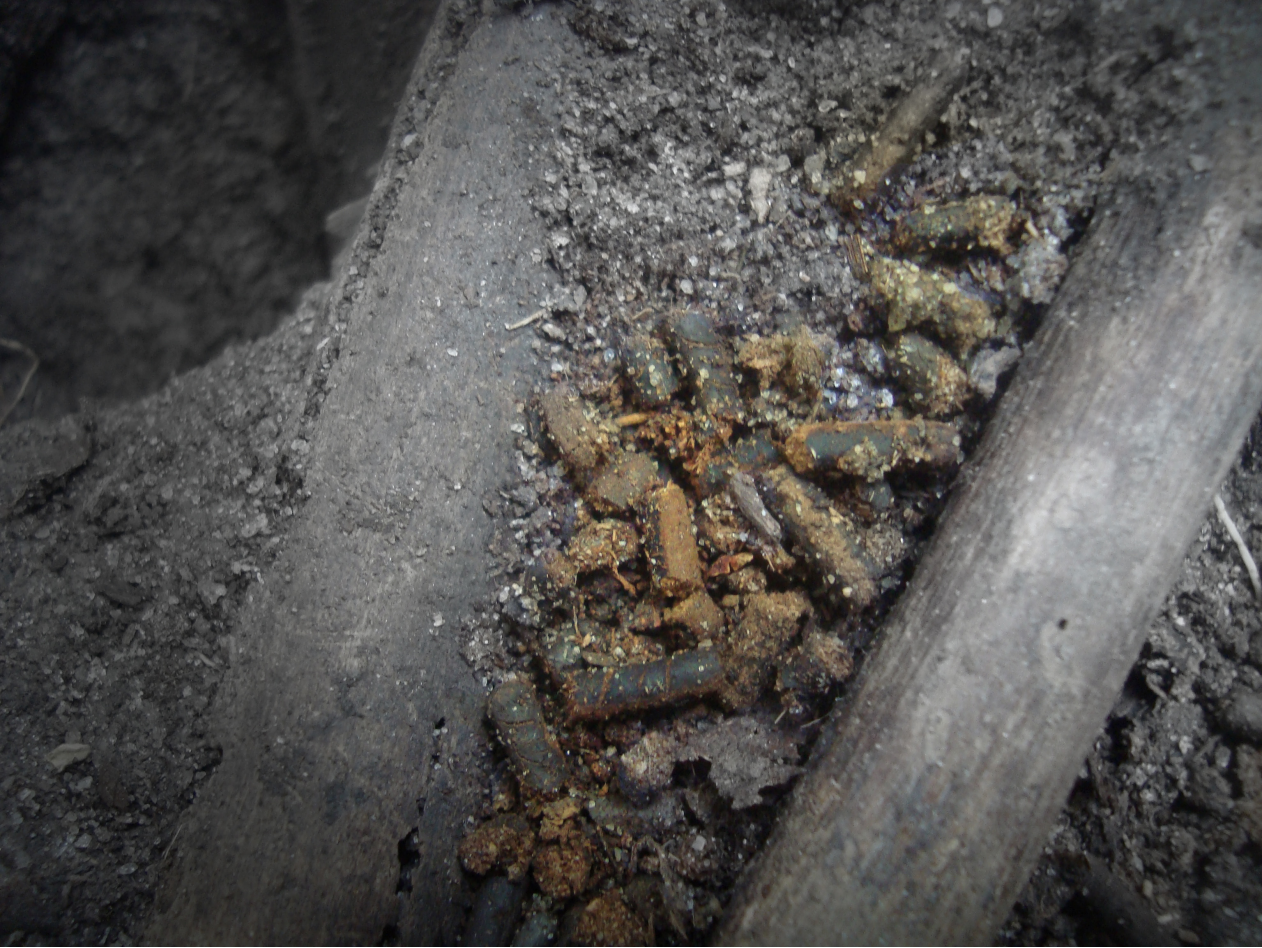
**Feces of a male specimen of *C. guanhumi*, according to the collectors**.

**Figure 5:**
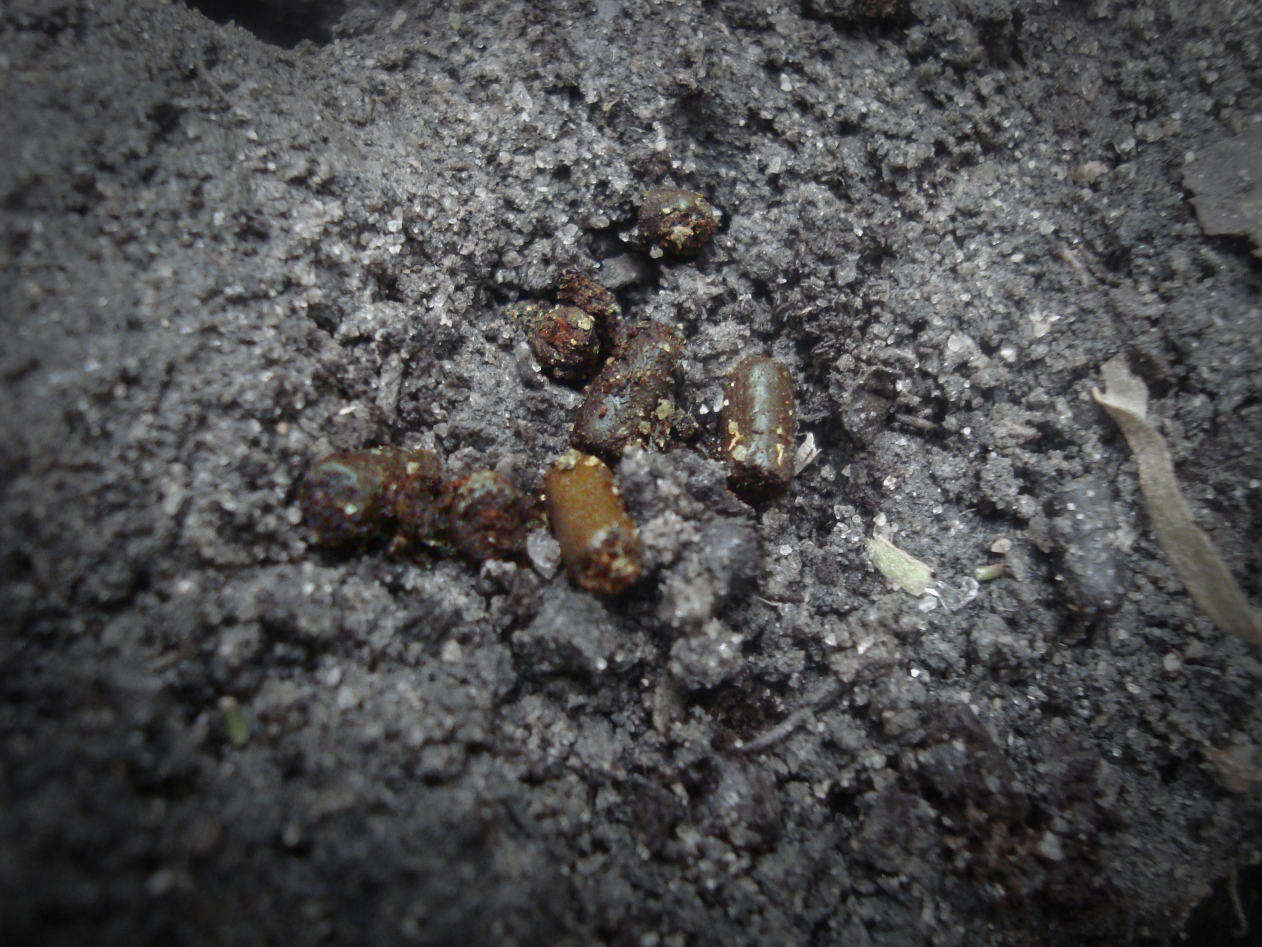
**Feces of a female specimen of *C. guanhumi*, according to the collectors**.

The crab-harvesters interviewed in Mucuri reported that they could distinguish between the tunnels of the Blue Land Crab males and females by observing the feces left at their entrances. A similar observation was reported by Takahashi [60] among Blue Land Crab harvesters living in four municipalities in the state of the Paraíba, Brazil. The interviewees in Mucuri indicated that the feces of the males are longer and thinner (Figure 4) than those of the females (Figure 5). The architecture of the tunnels can also indicate the sex of the animals, as the males construct long, inclined burrows, while the females construct straighter and more rounded burrows. The sexual differentiation of some species of crustaceans using morphological and behavioral characteristics has been reported by other crustacean harvesters [12,52,61-64].

The interviewees also noted that there was a relationship between the sizes of the animal and the sizes of their burrows. The Blue Land Crabs that these fishermen capture are initially selected using the criteria of tunnels size - for if the worker can fit his hand inside the burrow the animal is presumably large enough to be worth taking. The interviewees also noted that the females prefer softer soils and the males harder substrates. The harvesters also pointed out that individuals of *C. guanhumi *live solitary lives and spend most of their time within their galleries, leaving them only to feed, mate, or lay their eggs. According to Gifford [57], the upper section of the gallery is usually vertical, or very nearly so. Generally, only a single individual occupies a burrow; the males tend to be more territorial than females and will defend their burrows and small surrounding territories [48].

In terms of the trophic ecology of Blue Land Crabs, all of the interviewees agreed that this species consumed just about any resource available, including dead animals. This information corroborates Bright and Hogue [65] who noted that Blue Land Crabs are primarily herbivores that consumes vegetal material available in the sediment (e.g. the leaves of mangrove trees, flowers, etc.), although it can eat many other available items, such as small animals and decomposing plants. According to Hill [48], the basic food of this species is composed of red-mangrove (*Rhizophora mangle*) and white-mangrove (*Laguncularia racemosa*) leaves, as well as fruits and grasses.

The crab harvesters interviewed noted that there were two stages in the reproductive cycle of *C. guanhumi *during the year - the "andada" (walking about) (which is the mating period of these animals and occurs between January and March); and the "andada das fêmeas" (females walking about), or egg-laying phase between April and May. This information is consistent with the study undertaken by Botelho et al. [16] along the coast of the state of Pernambuco in which it was reported that the most intense reproductive period of these crabs occurred between December and February, and with Silva and Oshiro [66] who studied the reproduction of Blue Land Crabs in Sepetiba Bay in the state of Rio de Janeiro. According to a study undertaken by Gaião [67], the reproductive cycle of the Blue Land Crab initiates with the **"**andada" phase when the crabs leave their burrows in search of breeding partners. With the end of the "andada", the females begin their "desova" (egg-laying). It is worth noting that all of the interviewees stated they could distinguish between the mating and the egg-laying "andadas". This species reaches sexual maturity after approximately three or four years [48]. The crab harvesters recognize the sexual maturity of the Blue Land Crabs as being related to their size, when the animal has a carapace that is about 6 cm wide. This width is very close to that reported in the literature [51] as corresponding to sexual maturity (carapace 5.85 cm wide).

Another phase mentioned by the interviewees was ecdysis (called "descasca" by the crab harvesters). Although this phase does not occur simultaneously throughout the population, the interviewees reported that it was most common between July and August, when the animals are generally well-fed. According to Alves and Nishida [30] and Nunes [68], crustaceans such as *U. cordatus *stock their burrows with leaves and seal the tunnel mouth with mud in the period preceding ecdysis. The Mucuri crab harvesters stated that ecdysis in *C. guanhumi *can last one or two weeks, terminating between the months of September and October, when all of the animals will have completely molted. These animals then appear thinner due to the long periods spent inside their burrows during the molt. Nascimento [69] noted that this process allows the animals to increase in size, and is more frequent with animals in their larval and immature phases. Hill [48] observed that *C. guanhumi *will undergo up to 60 molts before reaching its adult phase, although these periods of ecdysis become less frequent with age. Blue Land Crabs (as well as the Mangrove Crab) are inappropriate for human consumption during this period and can cause serious collateral effects [55] and are popularly called "caranguejos de leite" (milk-crabs) due the secretion of a whitish liquid responsible for hardening the carapace [70]. This phenomenon occurs only in some species of crabs semi-terrestrial and terrestrial [71].

The biological cycles of many species that live in estuaries and mangrove swamps are heavily influenced by lunar cycles [30,72,73], and this might also be expected to affect human fishing activities [12,30,73,74]. All of the interviewees stated, however, that the lunar cycles and different tides did not interfere in the availability or harvesting of *C. guanhumi*. The only abiotic interference they mentioned was related to environmental temperatures, as these crabs do not react well to heat, preferring cooler temperatures and a rainy climate. The fishermen noted that when temperatures approached 40°C in the summer these crustaceans will rarely leave their burrows, making it more difficult to capture them. The optimal temperature for larvae development in this species in the laboratory was found to be between 25 and 30°C [48].

The crab gatherers reported that in addition to humans, Blue Land Crabs suffer natural predation from a mammal they call "Mão Lisa" (*Procyon cancrivorus*) (the Crab-Eating Raccoon), which eats both *C. guanhumi *and *U. cordatus*. According to these same fishermen, however, the relationship between them and the raccoon is quite neutral as they do not interfere with harvest production. This same species was also identified by the Blue Land Crab gatherers in the state of Paraíba as the most significant predator of *C. guanhumi *[60].

All of the crab harvesters interviewed affirmed that the sizes and the quantities of *C. guanhumi *stocks in Mucuri have been diminishing, principally due to pollution, destruction of the harvesting areas by fire, the conversion of mangrove areas to pasture for cattle, and over-exploitation by humans. Diminishing populations of *C. guanhumi *and other crustaceans in mangrove areas have likewise been reported by various other works [6,31,74,75]. Leite [51] stated that a large part of the population decline seen in *C. guanhumi *is a result of habitat losses.

According to the interviewees, there was a mass die-off of *U. cordatus *in 2005 that eliminated approximately 90% of the population of that crustacean in the area around Mucuri. This die-off apparently did not affect *C. guanhumi*, although it did provoke increased harvesting pressure on its populations. A similar situation was reported by Alves and Nishida [6,30] in the Mamanguape River estuary, PB, where a population crash of *Ucides cordatus *resulted in increased pressure on other crustacean species that had formerly been little used (putting their stocks at risk as well). The disease that caused the mortality of *Ucides cordatus *was called Lethargic Crab Disease [76] and was caused by the Ascomycota fungus, identified as "*Exophiala cancerae*" [77]; it apparently attacked *U. cordatus *almost exclusively.

### Local production practices

All of the interviewees agreed that the harvesting of Blue Land Crabs and other mangrove resources constituted their principal source of income. Seven of these men stated that they complemented their crab harvesting incomes by collecting other animals, such as the Mangrove Crab (4 interviewees) and by fishing (n = 2). One of the interviewees complemented his income by working in construction. This same sort of economic diversity was also reported in a study by Leite [51], with a number of Blue Land Crab collectors reporting that they also took on other jobs to add to the family income.

Most of the Blue Land Crab collection points are accessible by canoes ("bateras") that are propelled by rowing or by small gasoline motors. These boats are locally made, usually by the crab-harvesters themselves, and very few of the collection points can be reached on foot. The harvesters set aside three to five days a week to collect Blue Land Crabs, with daily efforts of from three to eight hours. As the collection areas ("apicuns") are located in the upper portions of the mangrove swamp [78], the harvesters do not have fixed working hours, although they are also not structured around tidal schedules - a situation quite distinct from that of other workers dependent on mangrove resources [73].

**Figure 6:**
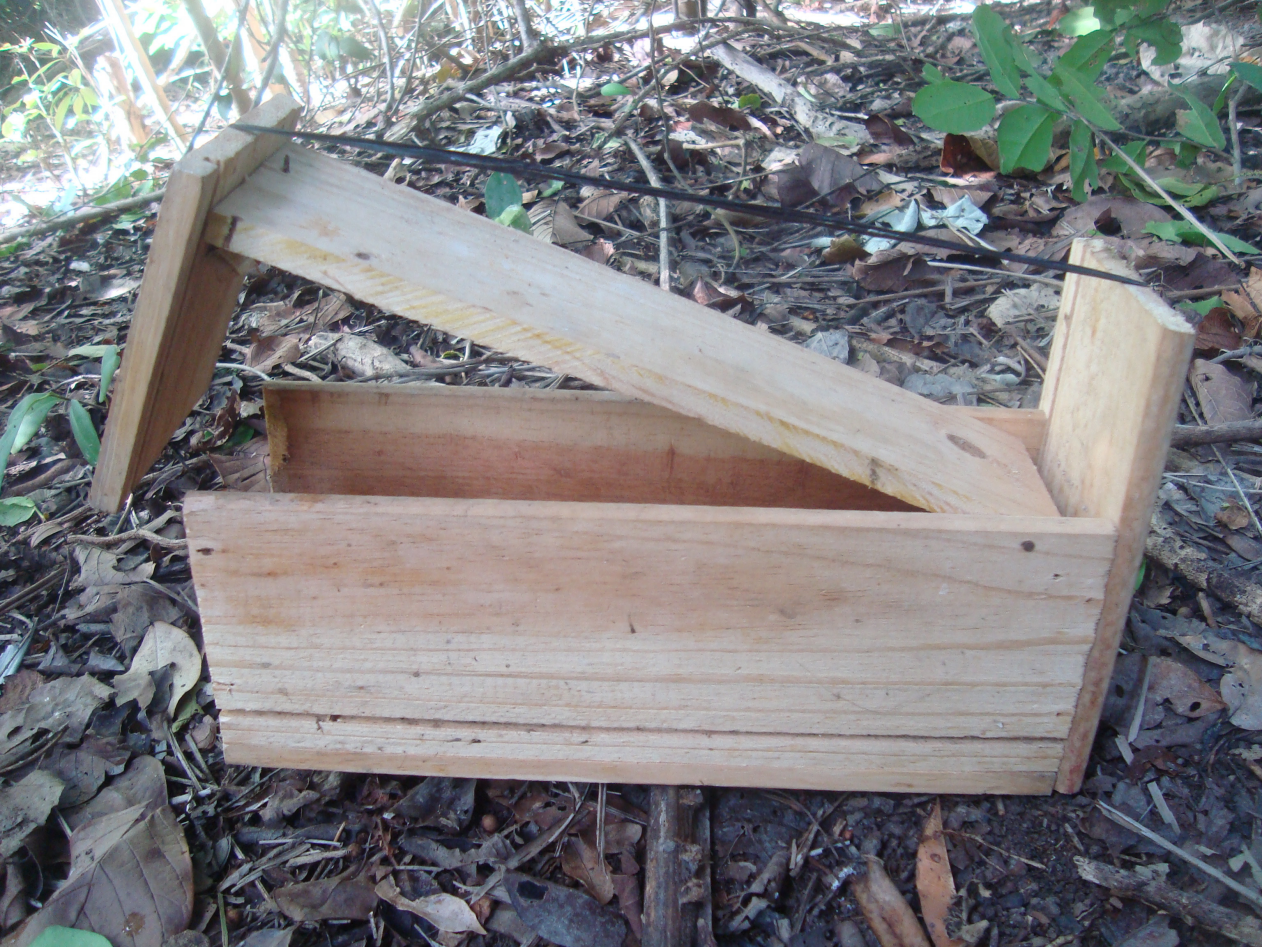
**Rat trap ("ratoeira") used to capture *C. guanhumi *until 2002, made of wood**.

**Figure 7:**
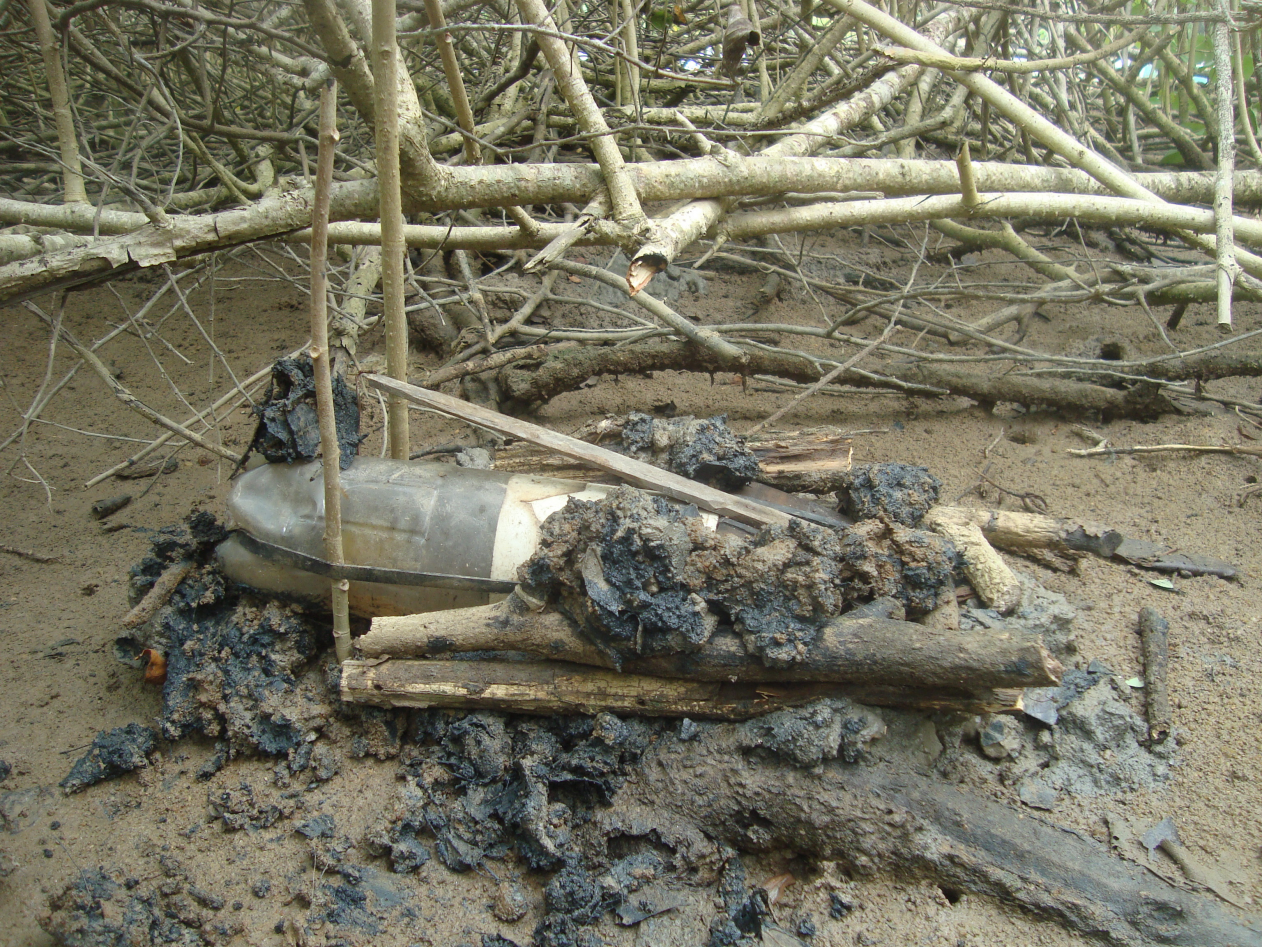
**Rat trap currently used by the collectors and made from plastic bottles and PVC pipe**.

The principal technique utilized for capturing Blue Land Crabs is a trap called a "ratoeira" (rat-trap) [15,79-81]. According to the interviewees, up until 2002 this device had been made exclusively from wood (Figure 6), but another crab-harvester from the state of Pernambuco introduced a new technique of making these traps using a plastic soda bottle fitted into a 6-inch PVC pipe and a wooden lid that was attached by rubber straps and triggered by a bait stick (Figure 7). When an animal (crab) triggers the trap by pulling on the bait, the door slams shut over the mouth of the tube. Other authors have described the use of this trap to capture the same species [12,51,82-84].

All of the interviewees have since substituted their wooden traps with plastic tube traps, in large part due to the facility in transporting them. The interviewees further explained that these traps do not injure the crabs before their sexes and sizes can be determined, and their use should not be considered a predatory practice. The use of these traps was, however, declared a predatory technique by federal authorities in 1994, although they were legalized again in 2006.

The harvesters carry between 15 and 50 traps on each outing that they set up near the openings of the crab dens and camouflage with leaves and mud (also fixing them to the sandy ground with sticks). The daily production of crabs is up to about 30 specimens per collector. The traps are generally set and then checked after one hour. Only two of the interviewees set their traps and then returned on the following day to collect them.

**Figure 8:**
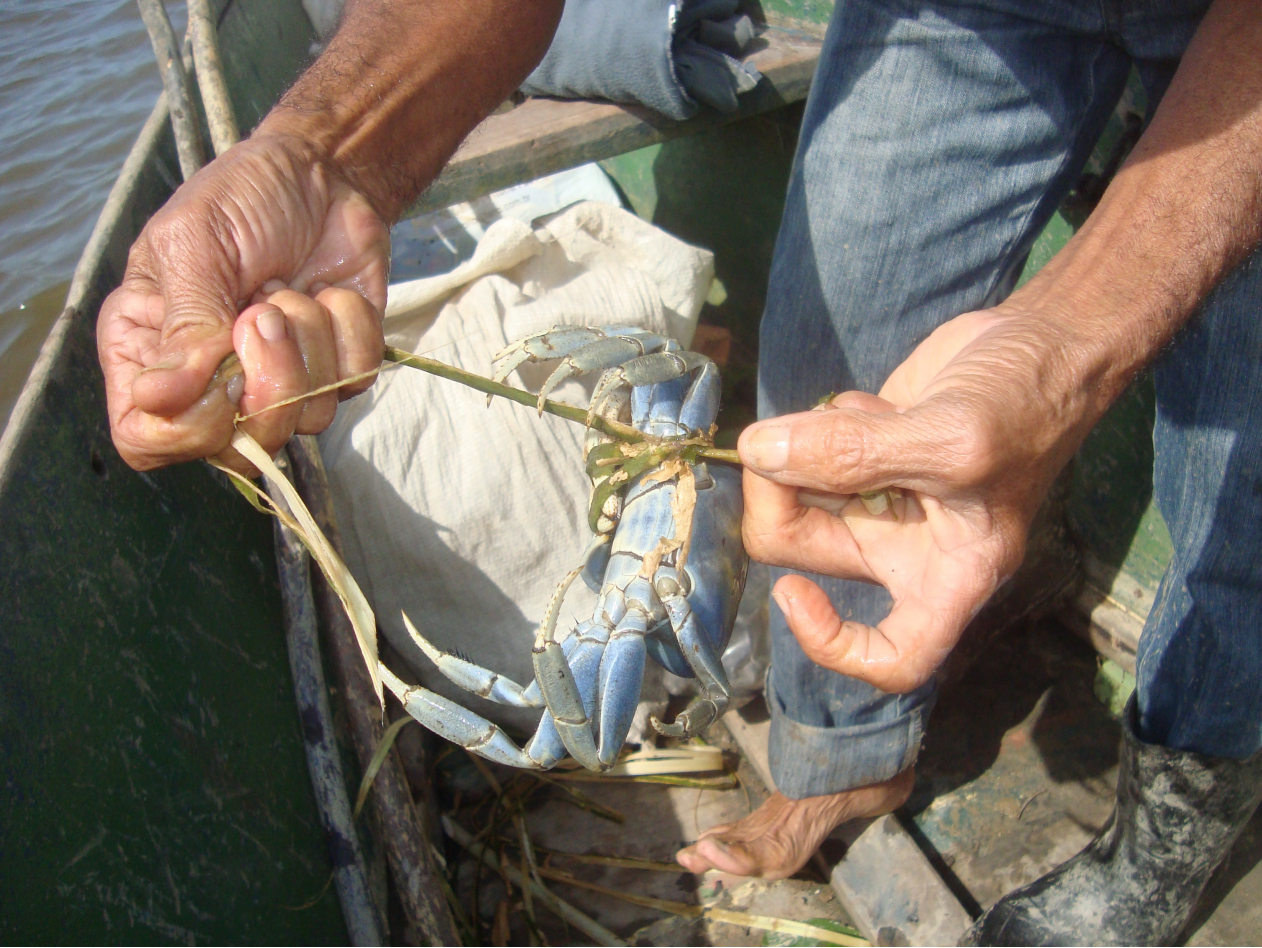
**Collector using the "imbira" twine to bind the land crabs that are picked**.

The bait used in the traps will depend on resources available at the moment. The baits most frequently used include: lemons (*Citrus *spp.), onions *(Allium cepa)*, corn (*Zea mays)*, dendê-palm fruits (*Elaeis guineensis*), jack fruit (*Artocarpus heterophyllus*), sugar cane (*Saccharum *spp.), genipap fruits (*Genipa americana*), pineapples (*Ananas comosus*), and "gravatá" (*Bromelia pinguin)*. The latter three are most favored as, according to the harvesters, sweeter and more aromatic baits are most successful. These types of bait have been reported in earlier publications [15,80,85]. The captured animals are subsequently bound with "imbira" (Figure 8) twine (which is made from strips of bark from the trunk of the Seaside Mahoe (*Hibiscus pernambucensis*) and then placed in large gunny sacks for transport.

At the end of the day the crabs are taken to the harvester's house and freed into storage pits called "chiqueiros" or "tanque de engorda", where they are kept and raised for up to 30 days (although they can be sold at any time, according to the necessities of the fishermen and/or demand). According to Gaião [67], Blue Land Crabs feed on essentially anything they find (such as the remains of dead animals and feces) and can thus accumulate toxins. The harvesters are aware of the details of their diets and for the first week after their capture they are fed only lemons to "clean" out their systems. During subsequent weeks they will be offered various other types of foods to gain weight. Gaião [67], Duarte [86], and Lima [87] likewise documented the fattening of these crabs while in captivity. The holding tanks may store up to an acceptable maximum of about 100 live animals. Generally, the smaller Blue Land Crabs are separated from the larger specimens and held in smaller tanks to avoid cannibalism. Cannibalism has frequently been observed under captive conditions [60] and it is believed that this species may exhibit this same behavior in their natural environment [48].

Most Blue Land Crabs are commercialized locally, being sold by the fishermen from their own houses to buyers that live in Mucuri. Very few crabs are sold to the owners of small seaside restaurants, as the city has apparently been loosing its former intense tourist appeal. The crabs are sold for between US$1.60 and $3.20 each, depending on their size. This value does not vary much, so that the fishermen tend to earn between US$230 and $650 per month throughout the year. Only three interviewees indicated that they sold their catches to buyers from neighboring cities.

According to Normative Instruction N° 90 DOU 06.02.2006, the Brazilian Institute of the Environment and Renewable Natural Resources (IBAMA) [88] is the responsible for regulating the capture, storage, transport, industrialization, and commercialization of *C. guanhumi *in northeastern Brazil.

When questioned about capturing *C. guanhumi *during the period when harvesting is officially prohibited (December to March) (according to Article 2° of regulation N.I. 90/2006), all of the interviewees indicated that they were aware of this non-harvesting period - although only eight of them knew the correct dates. Those did that did not know the correct dates of the prohibition period indicated that they did not follow this rule anyway because it was inadequate for the region and did not coincide with the egg-laying period of *C. guanhumi *in Mucuri. This situation demonstrates the necessity of regional studies that can use the knowledge of local fishermen to correctly determine the defense periods for crab reproduction and more adequately manage and conserve these species.

The interviewees stated that although they respected the legislation prohibiting the capture or sale of female crabs in the state of Bahia at any time of the year (Article 1° of N.I. 90/2006), it is not commercially feasible to just sell males. Additionally, the carapaces of the Blue Land Crabs sold in Mucuri have a minimum size of 6 cm, which demonstrates the circumvention of yet another federal regulation that prohibits capturing individuals of this species withcarapaces smaller than 7.0 cm (Article 4° of N.I. 90/2006). The interviewees noted that their preoccupation with regards to the sex of the crabs and the conservation of the species was not shared by the buyers (who were more interested in the sizes, and therefore the commercial values, of the animals).

## Conclusions

The results of the present work demonstrated that the community of crab harvesters in Mucuri retains a vast and important volume of knowledge about *C. guanhumi *that could subsidize both scientific studies and the elaboration of viable management and conservation strategies for this species. In light of the significant importance of this species in Brazil, ethnoecological studies should be encouraged as they aggregate additional information to scientific projects that can in turn be used to formulate plans for the sustainable use of this mangrove resource. IBAMA recently elaborated a national plan for managing economically important crustacean species, including Blue Land Crabs [5]. In addition to suggesting norms regulating the types of traps used to capture Blue Land Crabs, the plan points to the necessity of developing educational activities within fishing communities as well as more adequate monitoring and control of the mangrove swamp itself. As such, the plan recommends reductions in collections in this environment, with restrictions of human presence in the higher regions of the mangrove swamp above the high-tide line areas ("apicum") so that the galleries of young crabs will not be damaged or destroyed by foot traffic. It also recommends prohibiting the practice of commercializing isolated parts of these animals (claws). Additionally, the plan cites the necessity of the establishing specific extraction and exclusion areas. It will therefore be necessary to identify (through geo-processing technologies and physiographic characterizations) "apicum" regions (exclusion areas) and "restinga" sites (extraction areas with greater densities of adult individuals). It is hoped that our results will be useful in future revisions of the present plan and that it will incorporate the accumulated traditional knowledge and experience of populations that use these estuary resources.

## Competing interests

The authors declare that they have no competing interests.

## Authors' contributions

AMSF wrote all the parts of the manuscript, literature survey and interpretation and conducted the data collection. MMPT, RRDB, SRS and RRNA wrote some parts of the manuscript, ethnozoological data, literature survey, interpretation and revision of the manuscript. AMSF and MMPT analysis of taxonomic aspects. AMSF and SRS participated in data collection during the survey. All authors read and approved the final manuscript.
